# NOS1AP *O*-GlcNAc Modification Involved in Neuron Apoptosis Induced by Excitotoxicity

**DOI:** 10.3390/ijms160716560

**Published:** 2015-07-21

**Authors:** Liang Zhu, Tao Tao, Dongmei Zhang, Xiaojuan Liu, Kaifu Ke, Aiguo Shen

**Affiliations:** 1 Department of Neurology, Affiliated Hospital of Nantong University, the Jiangsu Province Key Laboratory for Inflammation and Molecular Drug Target, Nantong University, Nantong 226000, China; E-Mails: zhuliang_nt@163.com (L.Z.); nttaotao@ntu.edu.cn (T.T.); zdm@ntu.edu.cn (D.Z.); lxj@ntu.edu.cn (X.L.); 2 The Jiangsu Province Key Laboratory for Inflammation and Molecular Drug Target, Nantong University, Nantong 226000, China; 3 Department of Co-innovation Center of Neuroregeneration, Nantong University, Nantong 226000, China

**Keywords:** *O*-GlcNAc modification, NOS1AP, excitotoxicity

## Abstract

*O*-Linked *N*-acetylglucosamine, or *O*-GlcNAc, is a dynamic post-translational modification that cycles on and off serine and threonine residues of nucleocytoplasmic and mitochondrial proteins. In addition to cancer and inflammation diseases, *O*-GlcNAc modification appears to play a critical role during cell apoptosis and stress response, although the precise mechanisms are still not very clear. Here we found that nitric oxide synthase adaptor (NOS1AP), which plays an important part in glutamate-induced neuronal apoptosis, carries the modification of *O*-GlcNAc. Mass spectrometry analysis identified Ser47, Ser183, Ser204, Ser269, Ser271 as *O*-GlcNAc sites. Higher *O*-GlcNAc of NOS1AP was detected during glutamate-induced neuronal apoptosis. Furthermore, with *O*-GlcNAc sites of NOS1AP mutated, the interaction of NOS1AP and neuronal nitric oxide syntheses (nNOS) decreases. Finally, during glutamate-induced neuronal apoptosis, decreasing the *O*-GlcNAc modification of NOS1AP results in more severe neuronal apoptosis. All these results suggest that *O*-GlcNAc modification of NOS1AP exerts protective effects during glutamate-induced neuronal apoptosis.

## 1. Introduction

*O*-Linked *N*-acetylglucosamine (*O*-GlcNAc) is a highly dynamic post-translational modification which cycles on and off serine or threonine residues of numerous proteins [1,2]. The enzyme responsible for attaching *O*-GlcNAc to nucleocytoplasmic proteins is Uridine diphospho-*N*-acetylglucosamine: polypeptide β-*N*-acetylglucosaminyltransferase (*O*-GlcNAc transferase, OGT) [3], and the enzyme which is responsible for *O*-GlcNAc removal is β-*N*-acetylglucosaminidase (*O*-GlcNAcase, OGA) [4]. *O*-GlcNAc modification plays significant roles in many basic cell functions, such as cell morphogenesis, signal transduction, apoptosis and transcription [1,5,6,7]. Recently, more and more research indicates *O*-GlcNAc modification of proteins may be protective during cellular stress [8,9,10].

Nitricoxide synthase adaptor (NOS1AP), which is also named as carboxy-terminal PSD95-Dlg-ZO1 (PDZ) ligand of nNOS (CAPON), is highly enriched in brain [11]. It interacts with the nNOS PDZ domain through its C terminus [11]. NOS1AP has been implicated as an important role in many human diseases, such as neurodegeneration, stroke, schizophrenia, post-traumatic stress disorder and depression, autism, sudden cardiac death and long QT syndromes [12,13,14]. Glutamate-induced neuronal apoptosis is regarded as intermediate mechanism of many neuronal diseases, including neurodegeneration and stroke [15,16]. Glutamate is one of the main excitotoxicity neurotransmitters and excessive amounts of glutamate activate the NMDA receptor [17,18]. NMDAR-gated calcium influx leads to nNOS activation, triggering a series of reactions, finally activating p38MAPK and JNKs and leads to neuronal apoptosis [15,19]. While NOS1AP plays a critical part in excitotoxicity [20], the specific role of NOS1AP and the relationship between NOS1AP and nNOS during neurotoxity has not yet been clear as some believe NOS1AP is an inhibitor of nNOS function and some hold the opposite opinion that NOS1AP mediates nNOS signaling and contributes to NMDAR/nNOS dependent events [11,20,21]. In this study, we found that NOS1AP carries *O*-GlcNAc modification, and its *O*-GlcNAc protects neurons from apoptosis during excitotoxicity.

## 2. Results and Discussion

### 2.1. Excitotoxicity Induces Protein O-Linked N-Acetylglucosamine (O-GlcNAcylation)

**Figure 1:**
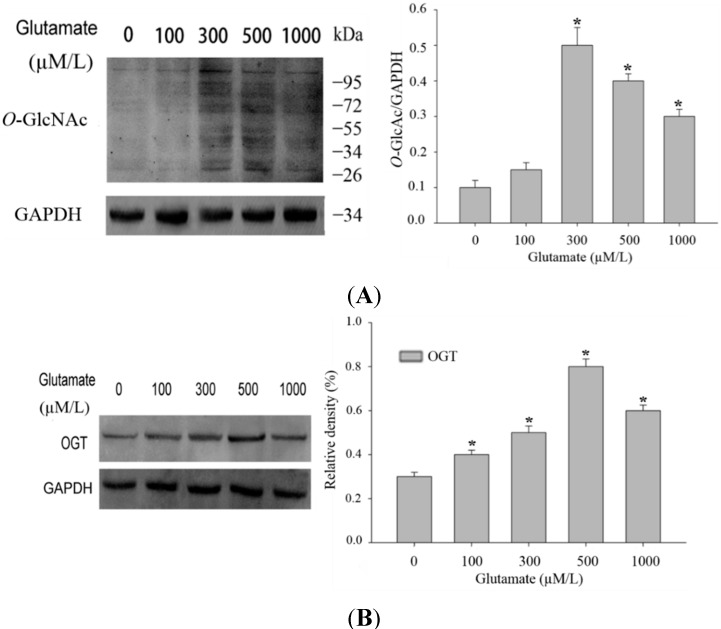
Alteration in *O*-GlcNAcylation of proteins after glutamate stimulation for 12 h in Rat pheochromocytoma (PC12) cells. (**A**) Cells were incubated with glutamate culture medium at concentration of 0, 100, 300, 500, 1000 μM/L separately. Proteins in cell lysates were subjected to sodium dodecyl sulfate (SDS)-polyacrylamide gelelectrophoresis (PAGE) and immunoblot analysis with anti-*O*-GlcNAc antibody (CTD110.6). The lane labeled 0, represent cells without glutamate stimulation, and the lanes labeled 100, 300, 500 and 1000 represent cells treated with 0, 100, 300, 500, 1000 μM/L glutamte respectively. The data are mean ± SEM. (* *p* < 0.05, significantly distinct from the group without glutamate treatment); (**B**) The expression level of OGT (*O*-GlcNAc transferase) was detected by Western blot. The data are mean ± SEM. (* *p* < 0.05, significantly distinct from the group without glutamate treatment).

It has been previously found that many forms of stress may increase protein *O*-GlcNAcylation. The increase of protein *O*-GlcNAcylation modification may be a protective response which is critical for cell survival. To determine whether protein *O*-GlcNAcylation would increase in the model of glutamate-induced excitotoxicity, we stimulated Rat pheochromocytoma (PC12) cells with different concentration of glutamate. As excepected, with a higher dose of glutamate applied, the *O*-GlcNAcylation of protein in PC12 cells increased (Figure 1A). As *O*-GlcNAcylation is specifically catalyzed by *O*-GlcNAc transferase (OGT), which transfers GlcNAc from UDP-GlcNAc donor onto proteins, we sought to analyse whether the increase of *O*-GlcNAc modification of total protein in the excitotoxicity model is due to alterations of OGT expression. Western blot results indicated that OGT expression level was indeed increased after mild stimulation of glutamate (Figure 1B). Thus higher levels of protein *O*-GlcNAc modification during glutamate-induced excitotoxicity may be mainly the result of the increased expression levels of O-GlcNAc transferase (OGT).

### 2.2. NOS1AP Is Modified with O-GlcNAc

**Figure 2:**
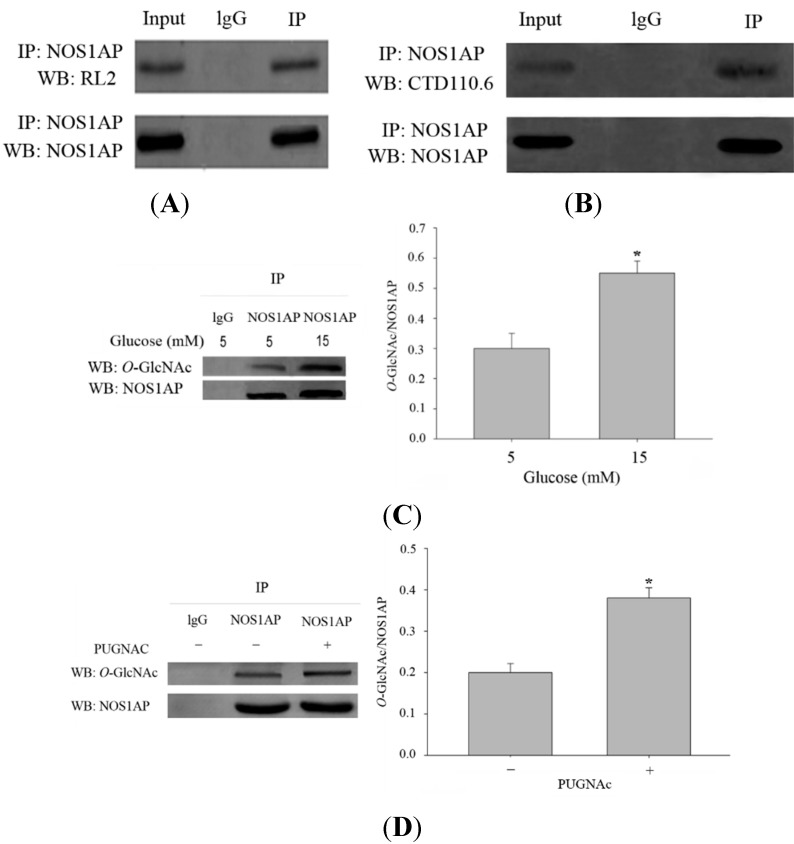
NOS1AP is modified with *O*-GlcNAc. Biochemical validation of *O*-GlcNAc on NOS1AP. (**A**) NOS1AP was immunoprecipitated from Human Embryonic Kidney 293 (HEK293) cells and subsequently blotted for *O*-GlcNAc using the RL2 antibody; (**B**) NOS1AP was immunoprecipitated from PC12 cells and subsequently blotted for *O*-GlcNAc using the CTD110.6 antibody; (**C**) *O*-GlcNAc of NOS1AP was enhanced when PC12 cells were cultured in high-glucose medium. The data are mean ± SEM. (* *p* < 0.05, significantly distinct from the 5 mM glucose group) and (**D**) *O*-GlcNAc of NOS1AP from PC12 cells was enhanced when stimulated with PUGNAC. The data are mean ± SEM. (* *p* < 0.05, significantly distinct from the group without PUGNAC treatment).

Abundant proteins in CNS carry the modification of *O*-GlcNAcylation. NOS1AP, a nNOS-associated protein, is highly enriched in brain and plays an important role in glutamate-induced neuronal excitotoxicity [20]. We sought to determine whether NOS1AP was modified with *O*-GlcNAc. Immunoprecipitated NOS1AP from embryonic kidney (HEK) cells 293 was probed with the *O*-GlcNAc-specific monoclonal antibody RL2. We successfully detected *O*-GlcNAc modification on NOS1AP (Figure 2A). Endogenous NOS1AP was also immunoprecipitated from PC12 cells and another *O*-GlcNAc antibody CTD110.6 was used in Western blots to confirm the *O*-GlcNAc attachment to NOS1AP (Figure 2B). However, considering the limitation of antibody RL2 and CTD110.6, (as RL2 can bind unmodified proteins and CTD110.6 will bind other glycans with terminal β-GlcNAc residues), we performed the following experiments to improve the specificity of *O*-GlcNAc modification. *O*-GlcNAc modification of NOS1AP was enhanced when incubated with high glucose compared with low glucose (Figure 2C). An increase of *O*-GlcNAc modification of NOS1AP was also observed when cells were incubated with *O*-(2-Acetamido-2-deoxy-d-glucopyranosylidenamino) *N*-phenylcarbamate (PUGNAc), an OGA inhibitor (Figure 2D). All results above confirmed that NOS1AP carries the modification of *O*-GlcNAc.

### 2.3. Mapping O-GlcNAc Sites on NOS1AP

**Figure 3:**
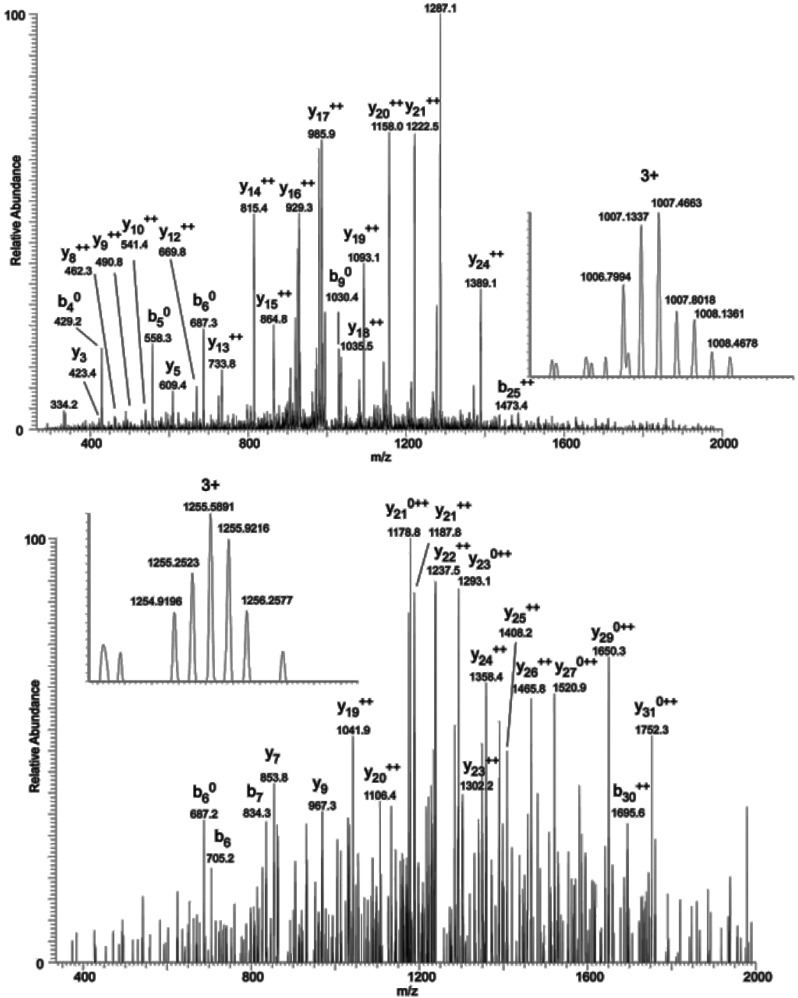

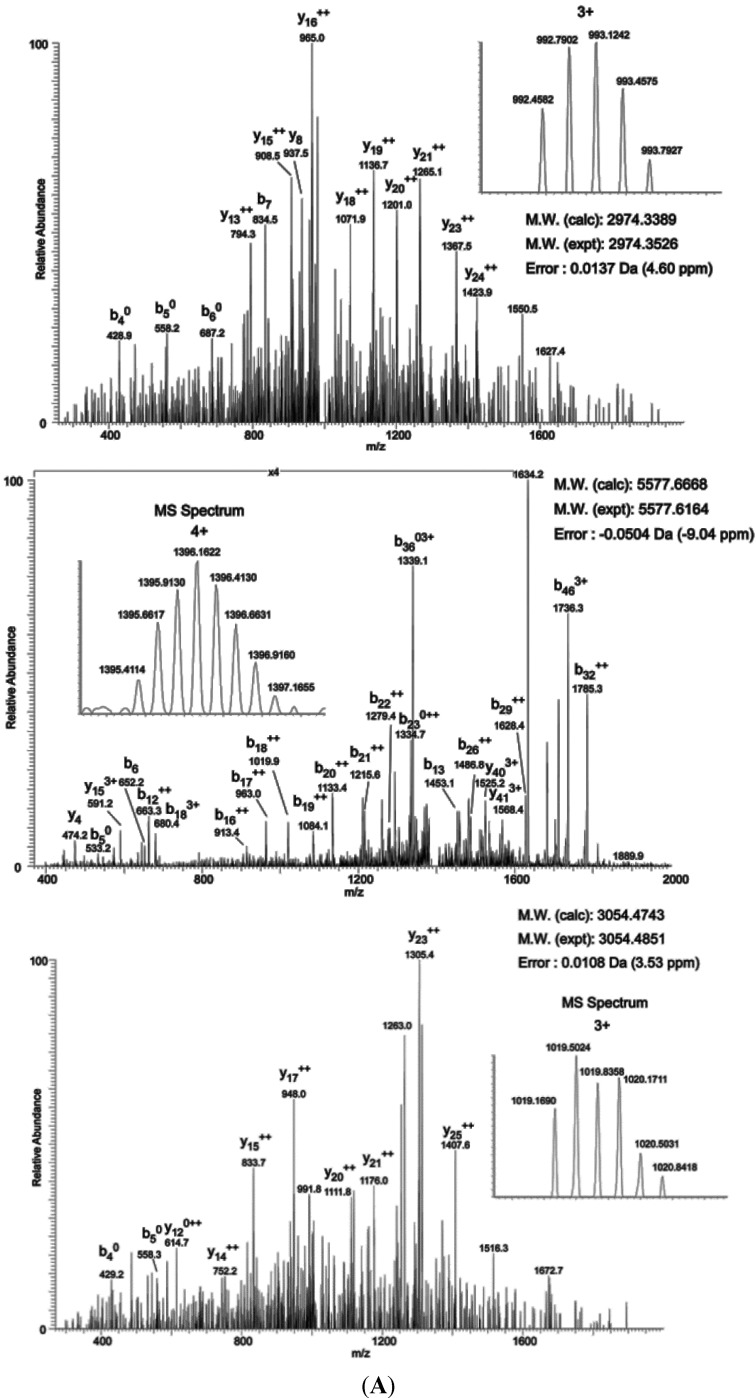

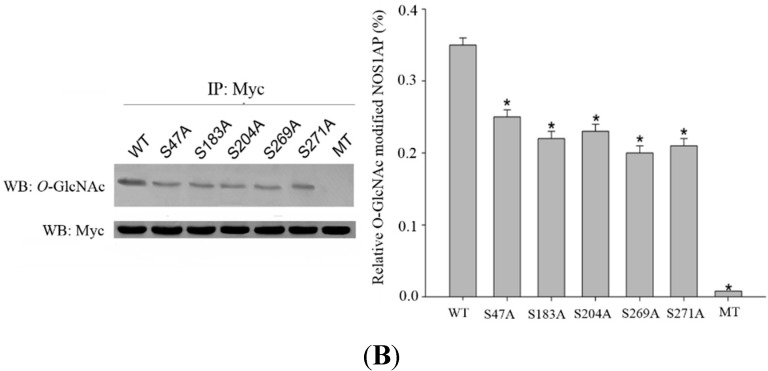
*O*-GlcNAc Sites on NOS1AP. (**A**) (ETD)-MS analysis indicated *O*-GlcNAc and phosphorylation modification sites; and (**B**) Different types of Myc-tagged NOS1AP were immunoprecipitated with anti-Myc beads, and immunoblotted for *O*-GlcNAc. WT represent wild type NOS1AP. Ser47, Ser183, Ser204, Ser269, Ser271 represent single site mutated NOS1AP. MT represent all *O*-GlcNAc sites mutated NOS1AP. The data are mean ± SEM. (* *p* < 0.05, significantly distinct from the WT group).

To map the glycosylation sites, NOS1AP was expressed in PC12 cells, immunoprecipitated and subjected to electron transfer dissociation (ETD)-MS analysis. The results of MS indicates that *O*-GlcNAc modification exists at Ser47, Ser183, Ser204, Ser269, Ser271 and phosphorylation modification exists at Ser269, Ser271 simultaneously (Figure 3A). To determine the specific *O*-GlcNAc sites, we created site-specific point mutants of NOS1AP and a mutant NOS1AP with all *O*-GlcNAc sites mutated. Exchange of Ser47, Ser183, Ser204, Ser269, Ser271 with Ala separately resulted in loss of *O*-GlcNAc modification and mutant NOS1AP with all *O*-GlcNAc sites showed no *O*-GlcNAc modification with CTD110.6 (Figure 3B). These results confirmed *O*-GlcNAc glycosylation at these sites.

### 2.4. Excitotoxicity Induces Glycosylation of NOS1AP

**Figure 4:**
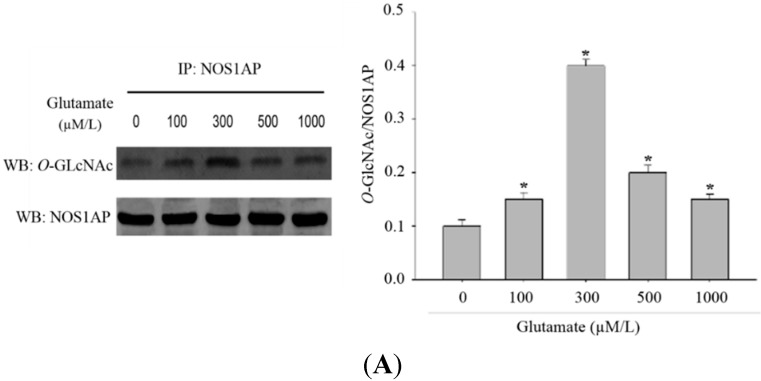

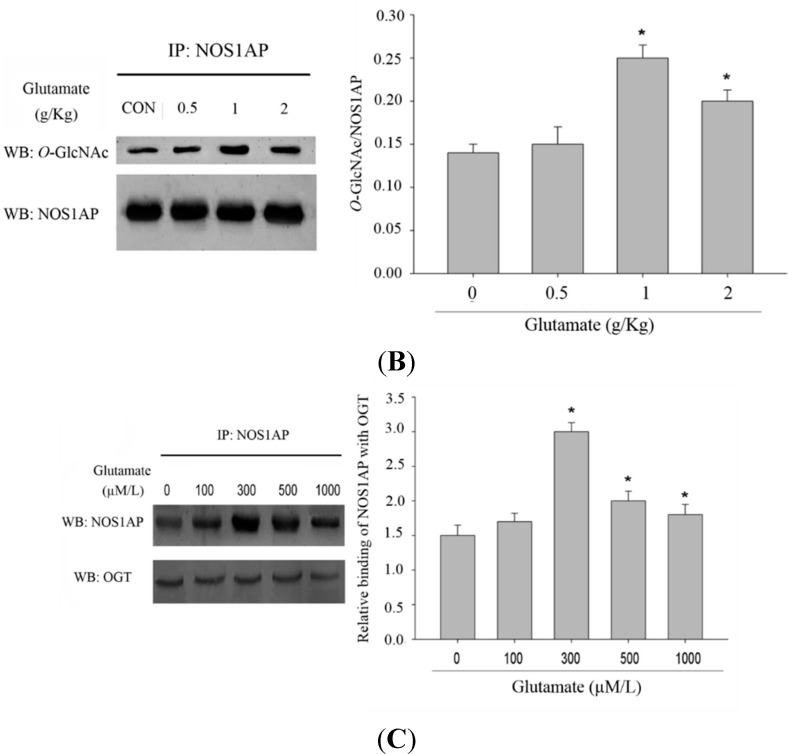
*O*-GlcNAc modification of NOS1AP was increased during glutamate-induced excitotoxicity. (**A**) PC12 cells were incubated with glutamate culture medium at concentrations of 0, 100, 300, 500, 1000 μM/L for 12 h, separately. The same amounts of NOS1AP were immunoprecipitated and immunoblotted for *O*-GlcNAc; The data are mean ± SEM. (* *p* < 0.05, significantly distinct from the group without glutamate treatment); (**B**) SD rats were treated with intraperitoneal injection of glutamate by 0.5, 1, 2 g/kg. The control group was treated with same volume of saline. After 2 h, NOS1AP was immunoprecipitated from brain tissue and immunoblotted for *O*-GlcNAc. The data are mean ± SEM. (* *p* < 0.05, significantly distinct from the control group) and (**C**) PC12 cells were incubated with glutamate culture medium at concentration of 0, 100, 300, 500, 1000 μM/L for 12 h separately. Same amounts of OGT were immunoprecipitated and immunoblotted for NOS1AP. The data are mean ± SEM. (* *p* < 0.05, significantly distinct from the group without glutamate treatment).

Previously, we demonstrated protein *O*-GlcNAcylation was increased in excitotoxicity models. As NOS1AP is strongly implicated in excitotoxicity signaling, we sought to determine whether the glycosylation of NOS1AP was changed during excitotoxicity events. As expected, when PC12 cells treated were treated with glutamate, glycosylation of NOS1AP was enhanced (Figure 4A). To verify the change of NOS1AP glycosylation in excitotoxicity, we conducted an animal model. We found that, with higher doses of glutamate injected, the *O*-GlcNAc modification of NOS1AP was higher in the brain tissue of rats (Figure 4B). We next determined whether the interaction between NOS1AP and OGT was enhanced in model of excitotoxicity. As predicted, with the same amount of endogenous OGT immunoprecipitated, more endogenous NOS1AP was blotted in excitotoxicity models (Figure 4C), which may partly explained the increase of NOS1AP glycosylation during excitotoxicity.

### 2.5. Glycosylation of NOS1AP Exerts Protective Effects through Affecting Its Interaction with nNOS (Neuronal Nitric Oxide Syntheses)

**Figure 5:**
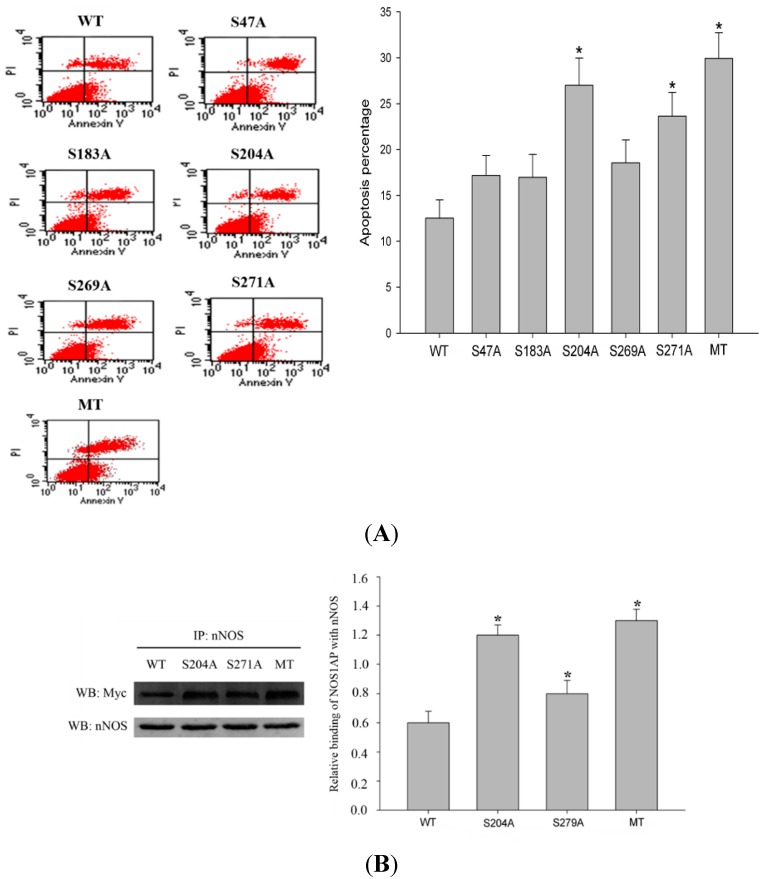
Glycosylation of NOS1AP exerts protective effects through affecting its interaction with nNOS. (**A**) PC12 cells were transfected with WT NOS1AP or *O*-GlcNAc sites mutated NOS1AP. Cells were stimulated with glutamate at concentration of 300 μM/L for 12 h before staining with FITC annexin V and propidium. Apoptosis was then evaluated by flow cytometry; (**B**) PC12 cells were transfected with Myc-tagged WT NOS1AP, S204A, S271A and all *O*-GlcNAc sites mutated NOS1AP. The same amount of nNOS was immunoprecipitated and immunoblotted for Myc. (* *p* < 0.05, significantly distinct from the WT NOS1AP transfected group).

Next, we determined whether the increase of NOS1AP glycosylation during glutamate stimulating would be protective or injurious. To assess the functional consequences of NOS1AP glycosylation on neuronal survival in excitotoxicity, we assayed apoptosis in PC12 cells expressing wild-type or S47A, S183A, S204A, S269A, S271A mutant NOS1AP. Western blot results confirmed similar expression levels of each mutant and wild type NOS1AP (data not shown). In contrast to cells expressing wild-type NOS1AP, cells expressing S47A, S183A, S204A, S269A, S271A mutant NOS1AP or MT (all *O*-GlcNAc sites mutated NOS1AP) showed more severe apoptosis, among which S204A, S271A and MT are most apparent (Figure 5A). Results above indicate glycosylation of NOS1AP exerts protective effects during excitotoxicity. To determine whether *O*-GlcNAc modification of NOS1AP exerts protective effect via nNOS, we checked interactions of nNOS with different forms of NOS1AP. Results showed that S204A, S271A and all glycosylation site mutated NOS1AP all showed increased interaction with nNOS (Figure 5B). These results indicate that *O*-GlcNAc modification of NOS1AP prevents its association with nNOS. Thus, we conclude that the increase of NOS1AP *O*-glycosylation during excitotoxicity decreases its association with nNOS, thus preventing the neurons from apoptosis.

### 2.6. Discussion

*O*-GlcNAc modification is a dynamic post-translational modification form, which has a close relationship with phosphorylation modifications [22,23]. Previous research has demonstrated that multiple forms of cell stress alter protein *O*-GlcNAcylation levels [24]. Among these stresses, some might promote protein *O*-GlcNAcylation through increasing glucose uptake and increasing intracellular levels of UDP-GlcNAc, the donor substrate for *O*-GlcNAcylation modification [24]. These modifications include reactive oxygen species, ultraviolet light, and ethanol *etc.* [24]. During the process, *O*-GlcNAcylated proteins function as regulators of cellular stress responses [24]. Studies have demonstrated that increasing *O*-GlcNAc levels alters the response of several key signaling pathways to stress, including nuclear factor (NF)-κB and mitogen-activated protein kinase (MAPK) pathways [25,26]. Accumulating data discovered that protein glycosylation increased tolerance to stress [9]. For example, it has been reported that inhibition of GFAT resulted in decreased *O*-GlcNAc levels and reduced cell survival after heat stress [8]. In addition, in rodent trauma-hemorrhage models, increasing *O*-GlcNAc levels during resuscitation attenuated the inflammatory response, increased organ perfusion and improved cardiac function [26]. Our study found higher *O*-GlcNAc modifications during glutamate stimulation, which is consistent with previous studies. The *O*-GlcNAc modification of proteins reached highest levels at a glutamate concentration of 300 µM/L. The up-regulation of *O*-GlcNAc modification may be a cell response against stress. When the applied glutamate concentration increased, however, the effect of up-regulation of *O*-GlcNAc modification was decreased. This might be due to a limited cell stress response reaction. When the stimulation is too intense, cell activity may decrease and result in less obvious up-regulation of *O*-GlcNAc modification. We next tried to figure out the mechanism of up-regulation of *O*-GlcNAc modification. We speculated that the increasing of *O*-GlcNAc modification might have resulted from changing amounts of UDP-GlcNAc, the substrate for *O*-GlcNAc modification, or maybe from changing levels or activity of related enzymes—OGT and OGA. To test our hypothesis, OGT expression level was measured and the OGT level was increased after glutamate stimulation.

Glutamate elicits neuronal response by binding to glutamate receptors. Glutamate receptors can be divided into two different groups: the fast-acting ligand-gated ion channels (iGluRs) and the slower-acting metabotropic receptors (mGluRs) [17,18]. Based on their physiological, pharmacologic, and molecular characteristic, the iGluRs can be further subdivided into three; categories: α-amino-3-hydroxy-5-methylisoxazole-4-propionic acid (AMPA), 10ainite (KA), and *N*-methyl-d-aspartate (NMDA) receptors [17,18]. Glutamate exerts its function mainly by activating the calcium-permeable ionotropic NMDAR [18]. Different subpopulations of the NMDAR may generate different functional outputs, depending on the signaling proteins directly or indirectly bound to its cytoplasmic tail [15]. NMDARs gate flux of calcium and sodium across the plasma membrane [27]. Sustained activation of the receptor leads to evident increased intracellular concentrations of both ions in neurons [14]. Among them, it is regarded that calcium/calmodulin activates nNOS. During the process, PSD95 is required to couple NMDAR-gated calcium in flux to nNOS activation [15,28,29]. NMDAR, PSD95, and nNOS form a ternary complex [28]. After nNOS is activated, NOS1AP was recruited to nNOS, affecting the interaction of nNOS with PSD95 [15,28].

In our research, we discovered the *O*-GlcNAc modification of NOS1AP was enhanced during glutamate-induced excitotoxicity, suggesting that glycosylation of NOS1AP has an important role in neuronal excitotoxicity. NOS1AP, is known as a ligand of nNOS and plays an important part in glutamate-induced neuronal excitotoxicity through its association with nNOS [11]. NOS1AP interacts with the nNOS PDZ domain through its C terminus [11]. The exact function of NOS1AP during excitotoxicity is not clear. It was previously thought that NOS1AP was an inhibitor of nNOS function, but no functional inhibition of nNOS has been reported [11]. Meanwhile, some studies suggest NOS1AP is a mediator of nNOS signaling and contributor to NMDAR/nNOS-dependent regulation of neuronal functions, such as glutamate-induced neuronal apoptosis [20,21]. We proposed that the function of NOS1AP during excitotoxicity is not simply inhibitory or as a mediator. NOS1AP may participate different signal pathways during excitotoxicity. Our research is mainly focused on the function of *O*-GlcNAc modification of NOS1AP in glutamate-induced excitotoxicity. We identified five *O*-GlcNAc sites on NOS1AP. To further investigate the specific function of *O*-GlcNAc modification on NOS1AP, we mutated *O*-GlcNAc sites with Ala to abolish the *O*-GlcNAc modification. With wild type NOS1AP and site-mutated NOS1AP transfected into PC12 cells respectively, we compared the different consequence in glutamate-induced neuronal apoptosis. As mentioned above, with *O*-GlcNAc modification abolished, the neuronal apoptosis was more significant than the wild type group. Among them, Ser204A, Ser279A, MT group showed more apparent neuronal apoptosis. Our mass spectrometry data previously suggested that NOS1AP may carry phosphorylation modification at Ser269 and Ser271. Thus, the exchange of Ser279 with Ala both abolished *O*-GlcNAc and phosphorylation modification at this site, while Ser204A mutant group induced more severe apoptosis without influencing phosphorylation sites. Considering that NOS1AP functions through binding to nNOS, we speculated that the *O*-GlcNAc modification of NOS1AP affects its association with nNOS. As expected, with *O*-GlcNAc sites on NOS1AP mutated, the interaction of NOS1AP and nNOS was enhanced, indicating that the *O*-GlcNAc modification of NOS1AP reduced its interaction with nNOS. Taken together, our results suggested that the *O*-GlcNAc modification of NOS1AP was enhanced during glutamate-induced excitotoxicity, thus preventing its association with nNOS, and finally protecting neurons from apoptosis. Further investigation is needed to explain how the *O*-GlcNAc modification of NOS1AP influences its association with nNOS and the exact role of the single *O*-GlcNAc sites on NOS1AP.

## 3. Experimental Section

### 3.1. Regents

PUGNAc was purchased from Toronto Research Chemicals Inc. (Toronto, ON, Canada). Cells were treated with 100 mM PUGNAc for 24 h before cell harvest or for 6 h before transfection. Wild-type or mutant NOS1AP-myc proteins were detected using anti-myc antibody (F-7425 for polyclonal and F-3156 for monoclonal, Sigma, Histon, WA, USA). CTD110.6, an antibody against *O*-GlcNAc was purchased from Covance (Princeton, NJ, USA). Antibodies against OGT (Sigma) was commercially available. Endogenous NOS1AP was detected with an anti-NOS1AP monoclonal antibody (Cell Signaling Technology Inc., Beverly, MA, USA). Antibody against GADPH and antibody RL2 were purchased from Santa Cruz Biotechnology (Santa Cruz, CA, USA).

### 3.2. Cell Culture and DNA Transfection

HEK 293, PC12 cells were cultured in DMEM (Gibco, Grand Island, NY, USA) supplemented with 10% foetal bovine serum, 100 U/mL penicillin, and 100 mg/mL streptomycin at 37 °C in 5% CO_2_. A rat NOS1AP expression vector with a C-terminal Myc and HIS epitope tag was purchased from Wuhan Sanying Company (Wuhan, China). Mutants of NOS1AP cDNA with Myc and HIS epitope, including the S47A, S183A, S204A, S269A, S271A mutants and MT (all *O*-GlcNAc sites mutated NOS1AP), were produced by PCR-based methods using a wild-type NOS1AP cDNA as template, followed by subcloning into the pcDNA3.1Myc-HIS mammalian expression vector (Invitrogen, Carlsbad, CA, USA). DNA plasmid was transiently transfected into PC12 cells using Lipofectamine 2000 (Invitrogen) according to the manufacturer’s instructions.

### 3.3. Western Blot

Samples were mixed with Laemmli Sample Buffer and boiled at 100 °C for 5 min. was performed with Bio-Rad (Hercules, CA, USA) 18% Criterion Tris-HCl Precast gel or “Any kDa” Mini-PROTEAN TGX Precast gel. Proteins were transferred onto Bio-Rad Trans-Blot Transfer Medium 0.2 µm nitrocellulose membrane, and blocked with 3% BSA, 0.02% Tween-20 in TBS for 1 h. The following antibodies were used: OGT; GAPDH; NOS1AP; nNOS; *O*-GlcNAc CTD110.6; *O*-GlcNAc RL2. After incubation, membranes were washed three times in 0.05% Tween-20 in TBS, and incubated in the appropriate secondary antibody linked to horseradish peroxidase for 1 h. Chemiluminescent signals were detected using Thermo Scientific SuperSignal West Pico solution (Thermo Fisher Scientific, Waltham, MA, USA).

### 3.4. Immunoprecipitation

Immunoprecipitation was performed with magnetic Dynabeads from Invitrogen, as per the manufacturer’s protocol. Nuclear extracts were obtained as described above. Antibodies were incubated in PBS or TBS with Dynabeads at room temperature. Proteins were eluted from the beads by boiling in Bio-Rad Laemmli Sample Buffer (Bio-Rad, Hercules, CA, USA) at 100 °C for 10 min.

### 3.5. Mass Spectrometry

PC12 cells were treated with *O*-(2-acetamido-2-deoxy-d-glucopyranosylidene) amino *N*-phenylcarbamate (PUGNAc, 100 µM, 6 h; Sigma) to inhibit β-*N*-acetylglucosaminidase and lysed in 1.5% SDS containing Complete™ protease inhibitor cocktail (Roche, Basel, Switzerland) and 5 µM PUGNAc. The lysate (7.5 mg) was diluted 1.5-fold, quenched with one volume of NETFS buffer (100 mM NaCl, 50 mM Tris-HCl pH 7.4, 5 mM EDTA, PIC, 5 µM PUGNAc) containing 6% (*v*/*v*) NP-40 and then was further diluted to 2 mg·mL^−1^ with NETFS buffer. The sample was passed over 400 µL of anti-NOS1AP affinity gel three times, washed three times with 10 mL of NETFS containing 1% (*v*/*v*) NP-40, washed twice with 10 mL of NETFS, eluted in 400 µL of 4% SDS, 100 mM Tris pH 7.9, and concentrated to a volume of 20 µL. After SDS-PAGE (4%–12% Bis-Tris gels), the NOS1AP band was excised and manually digested in-gel with trypsin or Glu-C at 37 °C. LC-MS analysis was carried out on LTQ mass spectrometer (Thermo, San Jose, CA, USA). Peptide mixture were separated on a C18 column. Mass spectrometric data were searched by SEQUST against NOS1AP protein sequence. The relevant searching parameters of mass range, intensity threshold, minimum ion count, precursor ion mass tolerance and fragment ion mass tolerance were set as 500–5000, 500, 15, 3.0 and 1.0 *m*/*z*, respectively. Trypsin or Glu-C was designated as the protease, and up to two missed cleavages were allowed. Carbamidomethylation of Cys was as a fixed modification, and phosphorylation (80.0 Da) and *O*-GlcNAcylation (203.2 Da) of Ser/Thr, and oxidation of Met (16 Da) were allowed as variable modifications. All the MS/MS spectra corresponding to possible *O*-GlcNAcylated peptides were manually inspected according to the following criteria: (1) the fragment ions matched were clearly above baseline noise; (2) contain sequential series of b- or y-ions; (3) contain the characteristic fragment peaks of Pro-directed, Asp-directed and His-directed fragmentation; (4) *O*-GlcNAc neutral loss. The identified *O*-GlcNAcylated peptides were further evaluated according to the principle of sequence tagging.

### 3.6. Animals and the Excitotoxicity Model

Male Sprague–Dawley rats (about 200 g) provided by the Department of Animal Center, Medical College of Nantong University were used in this study. They were kept in a temperature-controlled environment (21 °C) on a 12 h light–dark cycle. Rats were anesthetized intraperitoneally with sodium pentobarbital (50 mg/kg). Rats received intraperitoneal injections of 2 mL glutamate (0.5, 1 and 2 g/kg). Sham-controlled rats received an equivalent volume of saline. Experimental and sham-operated animals (*n* = 8 per group) were sacrificed after 2 h to extract the protein for immunoprecipitation and Western blot analysis. Experiments were carried out in accordance with the National Institutes of Health (NIH) Guidelines for the Care and Use of Laboratory and approved by the Chinese National Committee (No. 2014-10-213, 10 October 2014) to Use of Experimental Animals for Medical Purposes, Jiangsu Branch. All efforts were made to minimize the number of animals used and suffering.

### 3.7. Apoptosis Analysis by Flow Cytometry

The PC12 cells under certain treatment conditions were stained with FITC annexin V and PI using the BD Apoptosis Assay kit, and then evaluated for apoptosis by flow cytometry. PC12 cells transfected with different forms of NOS1AP were stimulated with 300 μM/L glutamate for 12 h before staining. The experiments were repeated with at least 5 replicates of each treatment.

### 3.8. Statistical Analysis

All experiments were repeated at least three times. All numerical data were described as mean ± SD. Data was analyzed using the two-tailed *t* test. A probability value of 0.05 or less was considered significant.

## 4. Conclusions

NOS1AP carries *O*-GlcNAc modification, and the *O*-GlcNAc modification determined its interaction with nNOS. The *O*-GlcNAc modification of NOS1AP protects neurons from apoptosis during excitotoxicity.
